# A gas exchange derived pulmonary capacitance to peak oxygen uptake slope threshold did not validate as a marker of early postoperative morbidity

**DOI:** 10.1016/j.bjao.2026.100582

**Published:** 2026-07-20

**Authors:** Zyad J. Carr

**Affiliations:** Department of Anesthesiology, Yale University School of Medicine, New Haven, CT, USA

**Keywords:** cardiopulmonary exercise testing, preoperative evaluation, preoperative risk stratification, submaximal cardiopulmonary exercise testing, validation

## Abstract

NCT05743673

Dear Editor

Rapid submaximal cardiopulmonary exercise testing has potential appeal in perioperative risk stratification because it may be more feasible than maximal exercise testing in older or frailer surgical candidates.^1^ In our derivation analysis, the gas exchange–derived pulmonary capacitance (GXCAP) to peak oxygen uptake (VO_2_) slope, measured as GXCAP to VO_2_, slope appeared to identify patients at increased risk of early postoperative morbidity.^2^ Specifically, a threshold of GXCAP-VO_2_ slope ≤ 25.2 was associated with postoperative morbidity defined by a the postoperative morbidity survey score (POMS) ≥1.^3^ We, therefore, sought to evaluate whether this prespecified threshold retained prognostic value in an independent internal validation cohort.

We analysed participants assigned to the validation cohort in our updated dataset. The primary endpoint was early postoperative morbidity, defined as POMS ≥1, selected a priori, to maintain continuity with the derivation analysis. The primary exposure was the previously identified derivation threshold, GXCAP-VO_2_ slope < 25.2. We first examined the unadjusted association between this threshold and POMS ≥1. We then fitted a prespecified multivariable logistic regression model adjusting for beta-blocker use and Physiological and Operative Severity Score for the enumeration of Mortality and Morbidity (POSSUM) operative severity.^4^ Results are reported as odds ratios with 95% confidence intervals.

The validation cohort included 150 patients, of whom 93 (62%) experienced POMS ≥1. Forty patients, or 26.7%, had a GXCAP-VO_2_ slope below the prespecified threshold of 25.2. Contrary to the derivation cohort finding, the event rate was nearly identical in patients below and above the threshold. Among patients with GXCAP-VO_2_ slope < 25.2, 25 of 40 developed POMS ≥1, corresponding to an event rate of 62.5%. Among those with GXCAP-VO_2_ slope ≥ 25.2, 68 of 110 developed POMS ≥1, corresponding to an event rate of 61.8%. The unadjusted odds ratio was 1.03, with a 95% confidence interval of 0.49 to 2.17 and *P*=0.939.

After adjustment for beta-blocker use and POSSUM operative severity, GXCAP-VO_2_ slope < 25.2 remained unassociated with early postoperative morbidity. The adjusted odds ratio (OR_adj_) was 0.89, with a 95% confidence interval of 0.41 to 1.96 and *P*=0.780. Beta-blocker use was also not independently associated with POMS ≥1 in this model, (OR_adj_=0.68, 95% CI: 0.34–1.37, *P*=0.280]). Unsurprisingly, the *post hoc* calculated POSSUM operative severity was associated with increased odds of early postoperative morbidity, (OR_adj_=1.15, 95% CI: 1.03–1.28, *P*=0.011]).

The discriminative value of the GXCAP-VO_2_ threshold alone was negligible, with an area under the receiver operating characteristic curve of 0.503. Using the threshold as a positive test gave sensitivity 26.9%, specificity 73.7%, positive predictive value 62.5%, and negative predictive value 38.2%. Thus, although the threshold classified approximately one-quarter of the validation cohort as higher risk, it did not meaningfully separate patients who did and did not develop early postoperative morbidity. The positive predictive value was therefore essentially the same as the overall event prevalence, indicating that the threshold did not enrich the probability of POMS-defined morbidity beyond the cohort baseline risk.

Sensitivity analyses supported the primary finding. Using clinically interpretable GXCAP-VO_2_ slope thresholds did not alter the findings. Thresholds of < 20, < 25, and < 50 were examined in adjusted logistic regression models including beta-blocker use and POSSUM operative severity. None was associated with POMS ≥1. A more stringent threshold of < 20 identified 16 participants and was not associated with increased risk (OR_adj_=0.51, 95% CI: 0.17–1.53, *P=*0.233).

These findings do not support external validation of the previously identified GXCAP-VO_2_ slope threshold for predicting early postoperative morbidity.^2^ The result is important because it illustrates a common challenge in perioperative biomarker and physiological signal development: a threshold that appears promising in derivation may not retain prognostic performance when applied to an independent cohort. This may reflect optimism in the derivation estimate, instability from dichotomising a continuous physiological measure, differences in case mix, or the multifactorial nature of early postoperative morbidity as captured by POMS.

Endpoints matter profoundly in perioperative outcomes research. Postoperative complications are heterogeneous, ranging from transient biochemical abnormalities to organ failure, reoperation, prolonged disability, and death. A composite endpoint such as POMS ≥1 is useful because it captures early morbidity across organ systems, but it may also group together events of very different clinical severity. As a result, a physiological marker may appear promising in derivation if the endpoint is broad, frequent, or variably ascertained, yet fail to validate when tested in an independent cohort.

For perioperative risk models to be clinically meaningful, endpoints must be clearly defined, objectively measured, consistently adjudicated, and clinically interpretable. This is particularly important when evaluating novel cardiopulmonary exercise testing (CPET)-derived metrics, where small differences in physiological signal may be overwhelmed by variation in surgical complexity, postoperative surveillance intensity, and how complications are classified. Binary outcomes such as any POMS morbidity provide statistical convenience, but may not reflect the burden, severity, or patient-centred importance of postoperative complications. The high event rate of POMS ≥1 did not mathematically preclude prediction but does make binary “any morbidity” endpoints less useful when it does not separate patients into meaningfully different absolute-risk groups. Future studies of submaximal CPET-derived markers should prioritise endpoints that distinguish minor postoperative deviations from clinically consequential morbidity. Candidate outcomes include specific pulmonary or cardiovascular complications, prolonged recovery, discharge destination, duration or persistence of morbidity, severity-weighted complication measures, and patient-centred recovery endpoints.

Therefore, negative validation of the GXCAP-VO_2_ threshold should be interpreted in the context of endpoint definition. The absence of association with POMS ≥1 does not necessarily exclude prognostic value for more specific or severity-weighted outcomes, such as pulmonary complications, cardiovascular events, prolonged recovery, or the Comprehensive Complication Index.^5^ Future validation studies should prioritise concrete complication endpoints that distinguish minor deviations from clinically consequential morbidity and should assess whether submaximal CPET markers predict not only the presence of complications, but also their severity, duration, and impact on recovery.

Validation cohort assessment is essential prior to clinical adoption of perioperative biomarkers or physiological thresholds. Our results should not be interpreted as evidence that submaximal CPET-derived indices lack value in perioperative assessment. Rather, they suggest that this single prespecified GXCAP-VO_2_ slope threshold, considered in isolation and after adjustment for beta-blocker use and operative severity, does not reliably identify patients at increased risk of early morbidity in the validation cohort. Future work should evaluate whether combinations of submaximal CPET variables, possibly using penalised regression or machine-learning approaches with appropriate internal and external validation, provide incremental prognostic value beyond established clinical and operative risk measures.

In summary, GXCAP-VO_2_ slope < 25.2 did not validate as a predictor of POMS ≥1 in an internal validation cohort. Early postoperative morbidity occurred at nearly identical rates above and below the threshold, and the adjusted association was null. POSSUM operative severity, rather than the GXCAP-VO_2_ threshold, was the only variable in the adjusted model that remained significantly associated with early postoperative morbidity.

## Author’s contributions

Zyad J. Carr contributed to study conception and design, acquisition of data, analysis, and interpretation of data, drafting and critical revision of the manuscript, final approval of the submitted version, and agreement to be accountable for all aspects of the work.

## Funding

Shape Medical Systems (White Bear Lake, MN) provided in-kind support for this study (equipment and disposable mouthpieces) and research assistant support.

## Declaration of interest

The author declares that they have no conflict of interest.
